# Transcranial Direct Current Stimulation to Augment Motor Imagery Training: A Systematic Review

**DOI:** 10.1111/ejn.70280

**Published:** 2025-10-28

**Authors:** Judith Bek, Amna Aziz, Nuala Brady

**Affiliations:** ^1^ School of Psychology University College Dublin Dublin Ireland; ^2^ Centre for Motor Control, Faculty of Kinesiology & Physical Education University of Toronto Toronto Canada

**Keywords:** brain‐computer interface, mental practice, motor imagery, neurorehabilitation, neurostimulation, transcranial direct current stimulation

## Abstract

Motor imagery training (MIT) is a widely used technique for motor learning and recovery. To optimize training outcomes, researchers have explored the integration of MIT with complementary approaches. One such approach is transcranial direct current stimulation (tDCS), which also shows promise as a method to enhance motor performance and neuroplasticity. This systematic review aimed to synthesize the current evidence on the synergistic effects of MIT combined with tDCS, with a specific focus on behavioral outcomes. Heterogeneous methods across 16 studies with 432 participants in total, including both healthy and clinical populations, yielded mixed results. Nonetheless, the potential of anodal tDCS applied over the primary motor cortex to augment the beneficial effects of MIT for motor performance in healthy participants is suggested by the current literature. The benefits of combining tDCS with MIT in brain‐computer interface (BCI) protocols with stroke patients were less clear, which may relate to population differences, timing of stimulation, or the similarity between outcome measures and trained tasks. Overall, small samples and heterogeneous methods limit interpretation of the findings of combined intervention studies, and further research should aim to measure both behavioral and neurophysiological outcomes in larger samples as well as examining longer‐term synergistic effects.

## Graphical abstract

Combining motor imagery training (MIT) with transcranial direct current stimulation (tDCS) may enhance motor outcomes compared to MIT alone. In healthy adults, concurrent anodal tDCS over primary motor cortex improved outcomes such as handwriting, postural control, and motor sequence learning. In stroke patients, most studies applied tDCS prior to MIT within brain‐computer interface (BCI) paradigms, showing less clear results. One study found no additional benefit of tDCS for pain reduction.

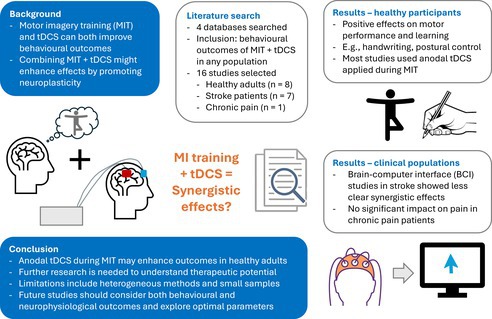

**Abbreviations**
ARAT: Action Research Arm Test
BCI: Brain computer interface
CRPS: Complex regional pain syndrome
DLPFC: Dorsolateral prefrontal cortex
EEG: Electroencephalogram
FMA: Fugl‐Meyer Assessment
GRASS: Guidelines for Reporting Action Simulation Studies
KVIQ: Kinesthetic and Visual Imagery Questionnaire
M1: Primary motor cortex
MI: Motor imagery
MIQ: Movement Imagery Questionnaire
MIT: Motor imagery training
NIBS: Noninvasive brain stimulation
PD: Parkinson's disease
PEDro: Physiotherapy Evidence Database
PRISMA: Preferred Reporting Items for Systematic Reviews and Meta‐Analysis
RCT: Randomized controlled trial
rTMS: Repetitive transcranial magnetic stimulation
SFTT: Sequential finger‐tapping task
SMA: Supplementary motor area
SRTT: Serial reaction time task
tDCS: Transcranial direct current stimulation
TMS: Transcranial magnetic stimulation

## 1 Introduction

Motor imagery (MI), also known as mental rehearsal or mental practice, refers to the process of mentally simulating a motor act in the absence of overt movement (Jeannerod 1994, 2001). MI includes internal representations of both visual and kinesthetic (imagined sensations associated with performing an action) elements of movement. Neuroimaging studies have indicated overlapping activations of brain areas such as the primary motor cortex (M1), supplementary motor area (SMA), and subcortical regions including the cerebellum, by both MI and physical execution (Hardwick et al. 2018). Given its potential to engage the motor system and promote motor learning and neuroplasticity, MI has gained recognition as an effective and accessible technique for training and rehabilitation, typically referred to as motor imagery training (MIT) (Schack et al. 2014).

Studies have shown the promise of MIT for motor performance and motor learning across various domains including sports, music, and medicine (Ladda et al. 2021; Lindsay et al. 2023; Schuster et al. 2011). The potential of MIT as a viable neurorehabilitation tool for patients with neurological conditions and movement disorders is evidenced by reviews of trials in Parkinson's disease (PD) (Caligiore et al. 2017; Kashif et al. 2023), stroke (Emerson et al. 2018; Guerra et al. 2017), and pain disorder (Bowering et al. 2013), as well as developmental coordination disorder (Scott et al. 2021). Research suggests that the benefits of MIT can be amplified through integration with other interventions such as physical therapy and virtual reality (Ferrero et al. 2021; Oostra et al. 2015). Noninvasive brain stimulation (NIBS) has also been explored for its potential to augment the effects of MIT, potentially enhancing neuroplastic effects. These approaches include transcranial magnetic stimulation (TMS) and transcranial direct current stimulation (tDCS). Although some studies using repetitive TMS (rTMS) in combination with MI have shown positive behavioral and neurophysiological outcomes (e.g., in stroke survivors; Zhang et al. 2024), the present review focuses on combining MI training with tDCS as a generally well‐tolerated and cost‐effective form of NIBS (e.g., Alonzo and Charvet 2021; Wansbrough et al. 2024) that might be suitable for a broad range of populations.

tDCS is a form of NIBS that modulates neuronal activity by altering the resting membrane potential. This modulation can enhance or inhibit spontaneous electrical activity, thereby facilitating neuroplasticity (Stagg et al. 2018; Woods et al. 2016). This technique involves delivering a weak electrical current, typically between 1 and 2 mA, between electrodes placed on the scalp, typically for a period of up to 30 min (Hassanzahraee et al. 2020; Martin et al. 2014). tDCS can be administered using two stimulation polarities: anodal or cathodal. Anodal stimulation, which delivers a positive charge, is generally associated with increasing cortical excitability and enhancing neuronal activity in the targeted brain region. This effect has been linked to improved learning and recovery processes (e.g., Cuypers et al. 2013). Conversely, cathodal stimulation, which delivers a negative charge, is typically associated with decreasing cortical excitability and suppressing neuronal activity, making it potentially useful for conditions such as epilepsy (e.g., San‐juan et al. 2015). Despite ongoing controversies surrounding the reliability and safety of tDCS, it is increasingly recognized for its tolerability, cost‐effectiveness, and feasibility as a clinical or home‐based therapy (Alonzo and Charvet 2021; Wansbrough et al. 2024). Benefits of tDCS for motor learning have been reported in healthy populations such as athletes (Chinzara et al. 2022). For patient populations, tDCS may offer a promising alternative or adjunct to conventional treatments for conditions including depression (Meron et al. 2015) and epilepsy (San‐juan et al. 2015; Sudbrack‐Oliveira et al. 2021). Evidence has also indicated potential benefits of tDCS as a tool for neurorehabilitation of patients with conditions including chronic pain, stroke, and Parkinson's disease (for reviews, see Bornheim et al. 2022; Broeder et al. 2015; Lloyd et al. 2020).

Similar to MIT, while tDCS as a standalone treatment may be beneficial, evidence suggests that when utilized in combination with other methods, its effectiveness can be enhanced (Chinzara et al. 2022). Extensive research has explored the effects of tDCS with physical therapy, consistently revealing positive outcomes for motor performance (Navarro‐López et al. 2024; Teixeira et al. 2020). While the exact mechanisms are not fully understood, tDCS appears to amplify cortical excitability associated with physical motor practice (Wang et al. 2021). Given that mental and physical motor training share overlapping neural substrates, it is plausible that combining tDCS with MIT could yield similar benefits (Foerster et al. 2013). Indeed, tDCS applied to M1 or premotor cortex has been found to enhance performance on mental rotation of hands, which is used as a measure of implicit MI (Date et al. 2015; Moghadas Tabrizi et al. 2019; for review, see Veldema et al. 2021), and stimulation of M1 and SMA affected performance of an explicit MI task (Yang et al. 2021). These findings suggest that tDCS might enhance MI and thereby boost the effects of mental practice.

Because of the potential neuroplastic and behavioral effects, as well as the relative safety, low cost, and noninvasive nature of both tDCS and MIT, their combination may provide a promising therapeutic intervention, particularly for older adults or patients who face challenges with traditional exercise regimes. An emerging body of literature has begun to explore the synergistic effects of combined tDCS and MIT. Individual studies have demonstrated potential benefits of this combined approach for motor performance and function in healthy participants (e.g., Saimpont et al. 2016) and clinical populations (e.g., Kashoo et al. 2022). Several reviews have reported the effectiveness of either MIT or tDCS independently and their integration with other interventions such as physiotherapy (Ladda et al. 2021; Navarro‐López et al. 2024; Schuster et al. 2011). A recent review (Zhang et al. 2024) examined the effects of combining MIT with NIBS (incorporating TMS as well as tDCS) for upper limb recovery in stroke survivors. Two further recent reviews (Lima et al. 2024; Mehrpour et al. 2024) explored the combined effects of tDCS with brain computer interface (BCI) technology that incorporates MI. However, the primary focus of these reviews was on the ability of the BCI mechanism to detect MI signals, rather than exploring the broader synergistic potential of MIT with tDCS. Consequently, this study aims to synthesize the current evidence regarding the efficacy of MIT with tDCS across both clinical and healthy populations, and to identify gaps to be addressed by future research. More specifically, this review examines whether combined MIT and tDCS enhances behavioral outcomes in different populations, and identifies the different protocols used for MIT, tDCS, and their combined application. We also assess the methodological quality of studies.

## 2 Methods

This systematic review was registered with the International Prospective Register of Systematic Reviews (PROSPERO; CRD42024533710) and was conducted in adherence with the Preferred Reporting Items for Systematic Reviews and Meta‐Analysis (PRISMA) statement (Page et al. 2021). The study protocol was approved by the research ethics committee at University College Dublin (2023‐30).

After registration, additional databases were added for a more comprehensive search process, and further data from the included papers were extracted based on the guidelines for reporting action simulation studies (GRASS, Moreno‐Verdú et al. 2024).

### 2.1 Search Strategy

A search strategy was developed to capture the relevant literature on the use of tDCS in combination with MIT. A comprehensive search string included three categories of key terms related to: 1) “transcranial direct current stimulation”, 2) “motor imagery training”, and 3) “intervention”, which were combined by utilizing the appropriate Boolean operators. A title and abstract literature search was systematically conducted across four electronic databases: PubMed, Web of Science, ProQuest, and Embase. The complete search strings are provided in the Supplementary materials (Table A). The final search was completed in May 2024, and the bibliographic references of two prior systematic reviews of combined BCI and tDCS (Lima et al. 2024; Mehrpour et al. 2024) were additionally reviewed. All identified records were then imported into Covidence for screening (Covidence Systematic Review Software, Veritas Health Innovation, Melbourne, Australia) (Kellermeyer et al. 2018). We did not include a systematic search of the grey literature due to the narrow, well‐defined scope of the review and the expectation that relevant high‐quality studies would be indexed in peer‐reviewed databases.

An initial search identified a heterogeneous set of studies, some of which focused on behavioral outcomes related to motor performance, while in others, the emphasis was on enhancing neural signals in BCI applications. A BCI is an artificial system that enables communication between the human brain and external devices through the translation of brain signals into commands that are captured using electroencephalogram (EEG) and typically modulated using MI (Blanco Mora et al. 2024). Some studies combining BCI with tDCS reported both behavioral and nonbehavioral (e.g., neural connectivity or cortical excitability) outcomes. The present review focused specifically on behavioral outcomes, which allowed for a more cohesive examination of measures relevant to combined MIT and tDCS across all included studies.

### 2.2 Eligibility Criteria

Studies were deemed eligible if they met the following inclusion criteria: (i) published in English, (ii) involved human participants, (iii) used any form of tDCS combined with any form of MIT as an intervention, and (iv) reported behavioral outcomes. In efforts to capture a broad range of literature, no restrictions were placed on the study publication date, population characteristics (e.g., age, sex/gender, or health status), or specific methods of MIT interventions or tDCS protocols (e.g., electrode placement, current intensity, or stimulation site). Studies were excluded if they were not in English, were performed with nonhuman subjects, or did not include behavioral outcomes (e.g., reported measures of brain activity only). Reviews, abstracts, protocols, and conference proceedings were also excluded.

### 2.3 Screening, Data Extraction, and Quality Assessment

Titles and abstracts of all identified studies were independently screened for eligibility by two reviewers using Covidence. Discrepancies were resolved through discussion within the research team. Full texts of the selected articles were then retrieved and further evaluated against the eligibility criteria. The full‐text screening was conducted by three reviewers who documented their assessments in a shared spreadsheet. Any discrepancies were discussed and resolved through consensus. After the identification of relevant studies, data extraction was carried out by one researcher. The following data were extracted: (i) study characteristics including author, year of publication, and study design, (ii) demographic characteristics including sample size, participant age, sex, and health status, (iii) details of tDCS protocols including duration, intensity, electrode placement, polarity, and number of sessions, (iv) details of MIT protocols including MI tasks and modalities, (v) details of combined intervention protocols, (vi) behavioral outcome measures, and (vii) main findings related to the outcomes of interest. In addition, we used the guidelines for reporting action simulation studies (GRASS, Moreno‐Verdú et al. 2024) to extract further details relevant to MI protocols (i.e., modality and visual perspective used in MI, specific MI instructions, measurement of adherence to MI protocol, assessment of MI ability, and previous familiarity with MI).

The methodological quality of the included studies was assessed using the Physiotherapy Evidence Database (PEDro) scale (Maher et al. 2003), a validated instrument that is commonly utilized in systematic reviews within similar fields (de Oliveira et al. 2022). The PEDro scale comprises an 11‐item checklist used to evaluate various aspects of study design and reporting. Each item is rated as “1” if the study meets the criterion and “0” if it does not. In line with standard practice (Maher et al. 2003), Question 1 is not included in the final PEDro rating; thus, each study receives a total score out of 10. The PEDro scale and study ratings are provided in Supplementary Materials (Table B).

### 2.4 Data Synthesis and Analysis

A narrative synthesis of the data was conducted, with results grouped by types of outcome measure. A meta‐analysis was not considered appropriate because of the heterogeneity of the protocols, populations, and outcome measures in the included studies.

## 3 Results

### 3.1 Study Selection

**FIGURE 1:**
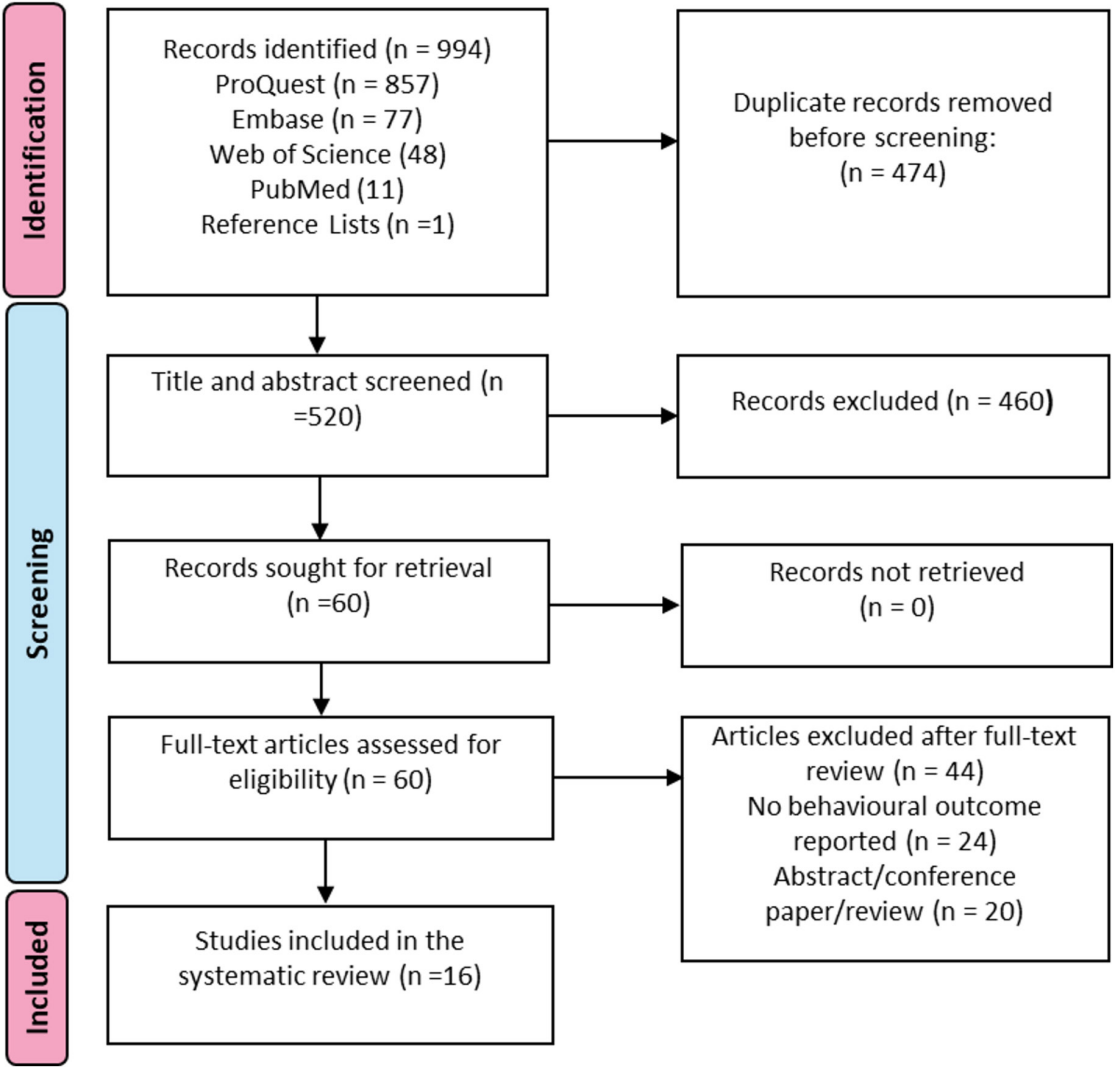
PRISMA flow diagram of study selection process.

The article selection process is illustrated in Figure 1. A comprehensive search across four databases identified a total of 993 records. One additional record was identified through manual inspection of bibliographic reference lists. Duplicates were initially removed by Covidence (*n* = 474) and were then checked by the researcher to verify duplicate status. Once title and abstract screening was completed, 60 full‐text articles were retrieved and examined. Following full‐text screening, 44 records were excluded because of not meeting criteria relating to publication type or inclusion of behavioral measures. The remaining 16 studies met the eligibility criteria and were included in the systematic review.

### 3.2 Study Quality

Methodological quality, as indicated by the PEDro scores for each included study, is detailed in Table B of the Supplementary Materials. The scores ranged from 5 to 9 out of 10 (M = 7.69), overall reflecting good to excellent methodological quality. The main risk of bias was the lack of allocation concealment, which was not present in many cases. Nonetheless, adequate randomization and blinding were generally implemented across studies, with a small number of exceptions (Hu et al. 2021; Sobierajewicz et al. 2019).

### 3.3 Study Design and Population Characteristics

The 16 included studies encompassed a range of design and population characteristics which are summarized in Table 1. The majority of studies (12/16) were randomized controlled trials (RCTs). Other studies were pseudo‐randomized (Metais et al. 2022; Saimpont et al. 2016), nonrandomized (Kasashima‐Shindo et al. 2015), and one was a randomized proof‐of‐concept study (Lagueux et al. 2018). Twelve studies employed a double‐blind design, two had a single‐blind design (Kasashima‐Shindo et al. 2015; Lagueux et al. 2018), and two did not specify their blinding status (Hu et al. 2021; Sobierajewicz et al. 2019).

**TABLE 1:** Characteristics of the included studies, listed in alphabetical order separately for studies on healthy and clinical populations.

Author (year)	Experimental design	Sample size (*n*)	Mean age (years)	Females (%)	Participant characteristics
Debarnot et al. (2019)	RCT	48	22.2	54.2	Healthy, right‐handed adults
Foerster et al. (2013)	Single group crossover RCT	18	23.2	88.9	Healthy, right‐handed adults
Metais et al. (2022)	Pseudo‐randomized	30	71.5	53.3	Healthy, right‐handed, older adults
Muller et al. (2024)	RCT	32	21	76.1	Healthy, right‐handed adults
Saimpont et al. (2016)	Pseudo‐randomized	36	25.1	66.7	Healthy, right‐handed adults
Saruco et al. (2017)	RCT	14	25.7	43.9	Healthy, right‐handed adults
Saruco et al. (2018)	RCT	16	20	56.3	Healthy, right‐handed and footed adults
Sobierajewicz et al. (2019)	RCT	48	24.5	70.8	Healthy, right‐handed adults
Ang et al. (2015)	RCT	19	54.1	26.3	Chronic stroke patients with moderate to severe upper limb disability
Chew et al. (2020)	RCT	19	56.4	5.3	Chronic stroke patients with moderate to severe upper limb disability
Hong et al. (2017)	RCT	19	54.1	26.3	Chronic stroke patients with moderate to severe upper limb disability
Hu et al. (2021)	RCT	19	54.1	11.9	Chronic stroke patients with moderate to severe upper limb disability
Kasashima‐Shindo et al. (2015)	Non‐RCT	18	50.8	27.8	Chronic stroke patients with moderate to severe upper limb disability
Kashoo et al. (2022)	RCT	56	59.1	25	Chronic stroke patients with upper limb disability
Lagueux et al. (2018)	Proof of concept RCT	22	47	63.7	CRPS 1 patients
Qi et al. (2019)	RCT	18	54.5	27.8	Chronic stroke patients with moderate to severe upper limb disability

Abbreviations: CRPS1 = complex regional pain syndrome type 1; RCT = randomized controlled trial.

Sample sizes ranged from 14 to 56 (mean *n* = 27) with a total of 432 participants across the 16 studies. Of these, 242 (56%) were healthy participants with no history of neurological disorders (Debarnot et al. 2019; Foerster et al. 2013; Metais et al. 2022; Muller et al. 2024; Saimpont et al. 2016; Saruco et al. 2017, 2018; Sobierajewicz et al. 2019), 168 (38.9%) were patients with a history of stroke presenting with moderate to severe motor impairments (Ang et al. 2015; Chew et al. 2020; Hong et al. 2017; Hu et al. 2021; Kasashima‐Shindo et al. 2015; Kashoo et al. 2022; Qi et al. 2019), and 22 (5.1%) were individuals diagnosed with Complex Regional Pain Syndrome (CRPS) type 1 (Lagueux et al. 2018). Two of the studies with stroke patients (Hu et al. 2021; Qi et al. 2019) also included age‐matched healthy controls; however, control data were only collected for neuroimaging and physiological outcomes, so these participants were excluded from the review. The proportion of female participants ranged from 5.3% to 88.9% (mean = 45.2%), and the mean age ranged from 20 to 71 years (mean = 40.4 years).

### 3.4 MIT Protocols and Outcome Measures

All MIT interventions (Table 2) were conducted in laboratory or hospital settings, with the exception of one which was conducted in the participants' homes (Lagueux et al. 2018). Eleven studies utilized MIT tasks, the majority of which closely aligned with an outcome measure of motor performance assessed before and after the intervention. For example, participants mentally practiced a handwriting task and were assessed on actual handwriting performance (Foerster et al. 2013), or practiced reaching and grasping tasks and were assessed on measures of upper limb functional mobility (Kashoo et al. 2022).

**TABLE 2:** Intervention details and outcomes of included studies, listed in alphabetical order separately for studies on healthy and clinical populations.

Author (year)	tDCS type and parameters	Stimulation site(s)	MIT task including modality, perspective, adherence checks	MI ability testing	Control conditions	Combined intervention protocol	Behavioral outcome(s)	Effects of tDCS +MIT on behavioral outcome
Debarnot et al. (2019)	Anodal, 2 mA/13 min	Right M1	Serial reaction time task using KMI and VMI (first‐person); adherence assessed by debrief after training.	Tested with MIQ‐3	PP + active or sham tDCS; MIT + sham tDCS	tDCS during MIT or PP for 3 consecutive days	Motor sequence learning	tDCS boosted effects of MIT on sequence‐specific learning more than PP after first session; tDCS enhanced consolidation of general motor skills in MIT group
Foerster et al. (2013)	Anodal, 2 mA/13 min	Right M1, right SMA, right premotor area, right cerebellum and left DLPFC	Writing words using KMI and VMI (first‐person); adherence NR.	Tested with KVIQ	MIT + sham tDCS	Six MIT sessions with tDCS (one per stimulation target) separated by 48 h; order of stimulation targets counterbalanced between participants	Handwriting	tDCS over M1 & DLPFC significantly enhanced effects of MIT on handwriting, particularly writing time and word size
Metais et al. (2022)	Anodal, 1.5 mA/13 min	Right M1	Finger sequence task using KMI and VMI (first‐person); adherence tested by task‐specific MI vividness ratings.	Tested with KVIQ‐10	MIT + sham tDCS	tDCS during 3 consecutive MIT sessions and retention session one week later	Motor sequence learning	tDCS did not significantly enhance effects of MIT vs. sham at any time point
Muller et al. (2024)	Anodal, 1.5 mA/13 min	Right M1	Finger sequence task using KMI and VMI (first‐person); adherence tested by task‐specific MI vividness ratings.	Tested with KVIQ‐10	MIT + sham tDCS	tDCS during 3 consecutive MIT sessions and retention session 1 week later	Motor sequence learning	tDCS did not significantly enhance effects of MIT vs. sham at any time point
Saimpont et al. (2016)	Anodal, 2 mA/13 min	Right M1	Repeated finger sequence task using KMI and VMI (perspective NR); adherence NR.	Tested with KVIQ‐10	MIT + sham tDCS or tDCS while reading a book	MIT performed for 15 blocks of 30 s each with 20 s rest; active or sham tDCS during MIT	Finger tapping	tDCS + MIT significantly enhanced effects on motor performance vs. MIT + sham and tDCS + reading
Saruco et al. (2017)	Anodal, 1 mA/12 min	Bilateral M1	Postural task using KMI; adherence tested by task‐specific vividness ratings of KMI.	Tested with KVIQ‐10	MIT + sham tDCS, manual precision game + sham tDCS	tDCS during MIT; condition order randomized; 1 week between sessions	Postural control	tDCS + MIT significantly improved performance vs. sham + MIT and sham + control task
Saruco et al. (2018)	Anodal, 1 mA/10 min	Bilateral M1	Postural task using KMI; adherence tested by task‐specific vividness ratings of KMI.	Tested with MIQ‐R	MIT + sham tDCS	MIT with different tDCS conditions: 1) before 2) during 3) both before and during; condition order counterbalanced; 1 week between sessions	Postural control	tDCS during MIT, but not before MIT, significantly improved performance vs. sham
Sobierajewicz et al. (2019).	Anodal or cathodal, 2 mA/15 min	Right M1	Motor sequence learning using KMI; adherence NR.	NR	MIT + sham tDCS or MIT with no stimulation (control)	tDCS during MIT for single session	Motor sequence learning	No significant improvement in sequence specific learning with anodal or cathodal tDCS vs. sham
Ang et al. (2015)	Anodal, 1 mA/20 min	Ipsilesional M1	MI‐BCI: visual feedback based on MI detection, robot‐assisted motion triggered by MI (1 h). Modality/perspective NR.	Screened for MI‐BCI ability	Sham tDCS before MI‐BCI	tDCS before MI‐BCI training for 10 sessions over 2 weeks	Upper limb motor function (FMA)	tDCS did not significantly enhance effects vs. sham
Chew et al. (2020)	Anodal, 1 mA/20 min	Ipsilesional M1	MI‐BCI: visual feedback based on MI detection, robot‐assisted motion triggered by MI. Modality/perspective NR.	Screened for MI‐BCI ability	Sham tDCS before MI‐BCI	tDCS before MI‐BCI training for 10 sessions over 2 weeks	Upper limb motor function (FMA)	tDCS group showed a trend towards greater improvement in motor function vs. sham
Hong et al. (2017)	Anodal, 1 mA/20 min	Ipsilesional M1	MI‐BCI: MI of reaching, robot‐assisted motion triggered by MI (40 mins). Modality/perspective NR.	Screened for MI‐BCI ability	Sham tDCS before MI‐BCI	tDCS before MI‐BCI for 10 sessions over 2 weeks	Motor function (FMA)	tDCS did not significantly enhance effects vs. sham
Hu et al. (2021)	Anodal, 1 mA/20 min	Ipsilesional M1	MI‐BCI: MI tasks with robotic feedback (1 h). Modality/perspective NR.	Screened for MI‐BCI ability	Sham tDCS before MI‐BCI	tDCS before MI‐BCI for 10 sessions over 2 weeks	Motor function (FMA)	tDCS did not significantly enhance effects vs. sham
Kasashima‐Shindo et al. (2015)	Anodal, 1 mA/10 min	Ipsilesional M1	MI‐BCI: MI of finger extension, robotic movement triggered by MI (45 mins). Modality/perspective NR.	Screened for MI‐BCI ability	MI‐BCI with no stimulation	tDCS before MI‐BCI for 10 sessions over 2 weeks	Upper limb motor function (FMA)	No significant difference between groups but tDCS group showed lasting improvement at 3 months vs. sham
Kashoo et al. (2022)	Anodal, 1.5 mA/30 min	Right M1	Upper limb movement sequence using VMI (perspective NR) and functional training; adherence NR.	Tested with KVIQ	Sham tDCS + MIT and upper limb functional training	Active or sham tDCS during MIT over 2 weeks with upper limb functional training	Upper limb motor function (FMA and ARAT)	tDCS + MIT with functional training significantly reduced upper limb impairment and disability vs. sham
Lagueux et al. (2018)	Anodal, 2 mA/20 min	Right M1	Graded MIT 10 min per session, 3 times per day, 6 times a week, for 6 weeks, including: 1) left–right limb discrimination task 2) imagined movements of the affected limb 3) mirror therapy. Modality/perspective NR. Adherence tested through home diaries.	NR	Graded MIT + sham tDCS	tDCS during graded MIT for 5 sessions per week in first 2 weeks then once per week for 4 weeks (total 14 sessions)	Pain severity measured by the BPI‐sf	tDCS + graded MIT did not significantly reduce pain vs. sham
Qi et al. (2019)	Anodal, 1 mA/20 min	Ipsilesional M1	MI‐BCI with robot‐assisted arm motion triggered by MI (1 h). Modality/perspective NR.	Screened for MI‐BCI ability	Sham tDCS before MI‐BCI	tDCS before MI‐BCI for 10 sessions over 2 weeks	Motor function (FMA)	tDCS did not significantly enhance effects vs. sham

Abbreviations: a‐tDCS = anodal transcranial direct current stimulation; BPI‐sf = Brief Pain Inventory short‐form; DLPFC = dorsolateral prefrontal cortex; EEG = electroencephalogram; FMA = Fugl‐Meyer Assessment; MI‐BCI = motor imagery‐brain computer interface; M1 = primary motor cortex; NR = not reported; PP = physical practice; SMA = supplementary motor area.

The MIT interventions involved mental practice using a diverse range of tasks. These included mentally rehearsing upper and lower limb activities such as sequential finger‐tapping (Metais et al. 2022; Muller et al. 2024; Saimpont et al. 2016), motor sequence learning (Debarnot et al. 2019; Sobierajewicz et al. 2019), balance and weight shifting (Saruco et al. 2017, 2018), handwriting (Foerster et al. 2013), reaching and grasping (Kashoo et al. 2022), and an upper and lower limb MI task using graded MIT (a structured approach involving several stages of MIT training for pain management) (Lagueux et al. 2018). Participants were instructed to use both visual and kinesthetic MI in five studies, visual MI in one study, and kinesthetic MI in two studies. Where visual MI was used, participants were usually instructed to take an internal (first‐person) perspective. Adherence to MI instructions was assessed in 6 studies, using self‐report ratings of task‐specific MI vividness, diaries, or poststudy debriefing.

In most of these studies, levels of MI vividness were assessed before or after the intervention. Vividness was tested using the Movement Imagery Questionnaire (MIQ; Hall and Martin 1997; Williams et al. 2012) in two studies and the Kinesthetic and Visual Imagery Questionnaire (KVIQ; Malouin et al. 2007) in six studies. However, details of participants' previous experience or familiarity with MI were typically not reported, and most studies did not report the specific instructions given to participants.

A further six studies, all of which were focused on stroke patients, utilized MIT within a BCI protocol (Ang et al. 2015; Chew et al. 2020; Hong et al. 2017; Hu et al. 2021; Kasashima‐Shindo et al. 2015; Qi et al. 2019). In these studies, participants underwent MI‐BCI screening to determine their ability to generate MI signals prior to the intervention and, in the majority, pretraining motor function was assessed by some variation of the Fugl‐Meyer Assessment (FMA; Fugl‐Meyer et al. 1975), an index of motor functioning, balance, sensation, and joint functioning in patients with poststroke paresis (muscular weakness). Most MI‐BCI studies followed a similar protocol, whereby the stroke‐affected upper limb was strapped to a robotic exoskeleton while subjects were instructed to imagine moving the affected arm, focusing on elbow and forearm movement (Ang et al. 2015; Chew et al. 2020; Hong et al. 2017; Hu et al. 2021; Qi et al. 2019). In one study, the arm was attached to a motor‐driven orthosis designed to facilitate finger movement, and participants were instructed to imagine extending their impaired fingers (Kasashima‐Shindo et al. 2015). In all of these studies, participants received visual feedback upon successful MI detection by the BCI system, which was measured via EEG. This feedback typically involved visual representations such as a moving cursor and served as a reinforcement of MIT by providing immediate confirmation that the BCI system correctly interpreted the brain signals associated with their imagined movements (Qi et al. 2019).

### 3.5 tDCS Parameters and Stimulation Targets

As shown in Table 2, tDCS parameters were relatively consistent across studies, although some differences were noted. Anodal tDCS was delivered in all of the studies, with the exception of Sobierajewicz et al. (2019) who compared both anodal and cathodal stimulation to sham or no stimulation. Stimulation was delivered through two sponge electrodes soaked in a saline solution, and in general, two electrode sizes were utilized across studies (25 or 35 cm^2^), with only one (Foerster et al. 2013) using a different electrode size (20 cm^2^). Stimulation intensity was 1 mA in eight studies, 1.5 mA in three studies, or 2 mA in five studies, and the current density of the active electrodes ranged from 0.04 to 0.06 mA/cm^2^.

The current was held constant for durations ranging from 10 (Kasashima‐Shindo et al. 2015) to 30 min (Kashoo et al. 2022), with the most common durations being 20 min (Ang et al. 2015; Chew et al. 2020; Hong et al. 2017; Hu et al. 2021; Lagueux et al. 2018; Qi et al. 2019) or 13 min (Debarnot et al. 2019; Foerster et al. 2013; Metais et al. 2022; Muller et al. 2024; Saimpont et al. 2016). A sham stimulation condition was typically included, in which electrode montages remained the same as for active stimulation conditions, except for one study that did not include a sham condition (Kasashima‐Shindo et al. 2015).

The targeted brain areas in all studies were chosen with the aim of stimulating regions associated with the relevant aspect of motor performance (predominantly upper limb tasks). The 10/20 international system for EEG was consistently followed to determine electrode placement. In studies involving healthy populations, the electrode positions varied slightly. Most commonly, the anode was placed over the primary motor cortex (M1), contralaterally to the nondominant hand, which in most cases was the right M1 (C3), while the cathode was placed contralaterally over the supraorbital region (Fp1). In one study (Sobierajewicz et al. 2019), the anode or cathode was placed over M1 contralaterally to the nondominant hand, depending on stimulation condition. In another study comparing stimulation of different brain areas, the anode was positioned over the right M1, right premotor area, right SMA, right cerebellar hemisphere, or left dorsolateral prefrontal cortex (DLPFC) in different sessions (Foerster et al. 2013). In two studies, to target both the left and right hemispheres, the anode was placed medially over M1 (Cz) and the cathode on the center of the forehead (Saruco et al. 2017, 2018).

In studies with patient populations, the anode was typically placed over the ipsilesional M1 (C3/C4), while the cathode was placed over the contralesional M1 (Ang et al. 2015; Chew et al. 2020; Hong et al. 2017; Hu et al. 2021; Qi et al. 2019) or the contralateral supraorbital area (Fp1) (Kasashima‐Shindo et al. 2015; Lagueux et al. 2018).

### 3.6 Combined Intervention Protocols

Details of combined intervention protocols and control conditions are provided in Table 2. As noted above, most studies were sham‐controlled, while some also employed control tasks with active and/or sham stimulation (Debarnot et al. 2019; Saimpont et al. 2016; Saruco et al. 2017), or MIT only without tDCS (Kasashima‐Shindo et al. 2015; Sobierajewicz et al. 2019). In all of the non‐BCI studies, tDCS was delivered concurrently with MIT. In one of these studies, tDCS before MIT was also investigated (Saruco et al. 2018). Combined MIT and tDCS interventions ranged from a single experimental session (Saruco et al. 2017) to 14 sessions over 6 weeks (Lagueux et al. 2018).

In MI‐BCI studies, participants received 10 sessions of the intervention over 2 weeks. The majority of these interventions comprised 20 min of active or sham tDCS followed by 40–60 min of MI‐BCI training including evaluation and rest periods. In one MI‐BCI study, participants received 10 min of active stimulation or no stimulation (Kasashima‐Shindo et al. 2015).

### 3.7 Study Findings

#### 3.7.1 Adverse Effects

Interventions were generally well tolerated, although some participants reported side effects of tDCS, such as local discomfort at the stimulation site (itching, burning), slight reddening, a temporary increase in pain (in patients with CRPS), or headaches (Kashoo et al. 2022; Lagueux et al. 2018). Some studies reported little to no adverse effects of tDCS (Chew et al. 2020; Kasashima‐Shindo et al. 2015; Metais et al. 2022; Saimpont et al. 2016), while the remaining 10 studies did not provide information on adverse effects.

#### 3.7.2 Motor Performance in Healthy Populations

Four studies investigated the effects of tDCS with MIT on sequential finger/hand task performance using the nondominant hand, yielding mixed results. The earliest of these studies (Saimpont et al. 2016) investigated effects on a complex eight‐item sequential finger‐tapping task (SFTT) in young adults. Results indicated that 13 min of MIT (imagining the SFTT) combined with anodal tDCS over M1 led to a significantly greater increase in the number of correctly performed sequences both immediately after MIT and 90 min later, compared with anodal tDCS alone or MIT alone. Other studies found less promising results. For example, two later studies that employed similar tasks with young adults (Muller et al. 2024) and older adults (Metais et al. 2022) demonstrated that after three consecutive sessions, motor sequence learning improved with MIT, but anodal tDCS did not further enhance these effects (i.e., there was no significant difference between sham and active tDCS groups). Sobierajewicz et al. (2019) similarly demonstrated that, while MIT alone influenced motor sequence learning, the addition of anodal tDCS did not increase sequence‐specific learning effects in terms of either speed or accuracy. Although nonspecific learning was increased after anodal tDCS compared to a no‐stimulation control group, effects were not significantly different between anodal, cathodal, and sham stimulation conditions, so a placebo effect could not be ruled out.

Debarnot et al. (2019) examined implicit motor sequence learning in young healthy participants through both physical practice with active or sham tDCS and MIT with active or sham tDCS. Performance of a serial reaction time task (SRTT) was examined in terms of general motor skills and sequence‐specific skills. Results indicated that anodal tDCS over M1 enhanced motor performance significantly more when combined with MIT than when combined with physical practice during the initial acquisition phase. While both physical practice and MIT groups benefited from anodal tDCS compared to sham, the combined effect of anodal tDCS with MIT consistently led to greater improvements and better skill consolidation. Specifically, significant performance gains in sequence‐specific skills were observed with MIT and tDCS on the first day. Consolidation of sequence‐specific skills was found with MIT but not physical practice, although this was not further influenced by tDCS. Moreover, although initial acquisition of general motor skills was not enhanced by tDCS in either the physical practice or MIT groups, consolidation of general motor skills showed delayed enhancement of learning by tDCS only in the MIT group.

Foerster et al. (2013) found that anodal tDCS significantly enhanced motor performance benefits induced by MIT, as evidenced through reduced writing time and improved legibility in a handwriting task with the nondominant hand, compared to MIT with sham stimulation. These effects were observed following stimulation of M1 and DLPFC, but not other brain areas such as the cerebellum, which conversely diminished aspects of handwriting performance.

Two studies by the same group provided evidence for improved postural control following combined tDCS and MIT. Saruco et al. (2017) used a complex postural task that required participants to shift their center of pressure to validate 16 targets in eight directions without lifting their feet or losing balance. Two experimental conditions in which participants imagined the postural task while receiving anodal or sham tDCS over M1, and one control condition in which participants played a manual precision interactive game while receiving sham tDCS, were compared. Although a significant decrease in the time required to perform the postural task was observed after a single session (12 mins) of MIT with sham tDCS, performance gains were greater when MIT was coupled with anodal tDCS over M1 compared to MIT with sham tDCS or the control task. This improvement was especially pronounced for tasks involving more demanding postural adjustments. Specifically, forward movements benefited from both MIT conditions, while backward movements showed significant improvement only in the MIT condition with active anodal stimulation. Saruco et al. (2018) employed a similar task with the addition of further conditions involving tDCS either before MIT or both before and during MIT. Reinforcing the previous findings (Saruco et al. 2017), significant postural improvements were found following anodal tDCS over M1 during MIT. However, no improvement was observed with tDCS before MIT or before and during MIT.

#### 3.7.3 Motor Recovery in Patient Populations

Some promising results of combined MIT with tDCS in patient populations have been reported. For example, Kashoo et al. (2022) showed that anodal tDCS over the ipsilesional M1 combined with MIT and functional training, compared to the same training with sham tDCS, reduced upper limb impairment and disability (FMA and Action Research Arm Test [ARAT]) among patients with chronic stroke, with improvements reaching clinically significant levels in the active tDCS group. However, pain disorder patients did not show a significant reduction in pain following graded MIT combined with anodal tDCS over M1 compared to graded MIT with sham tDCS (Lagueux et al. 2018). Although pain disorder patients showed improvements in kinesiophobia (fear of movement), pain catastrophizing, and anxiety after 6 weeks of treatment, these improvements were not maintained at 1 month after intervention and did not reach a clinically significant difference (Lagueux et al. 2018).

Six studies investigated the combined effects of MI‐BCI and anodal tDCS over the affected M1 on motor performance in stroke patients with moderate to severe impairments. While two studies found improvements in the upper extremity subscale of the FMA across both MI‐BCI with active tDCS and MI‐BCI with sham/no stimulation groups, Chew et al. (2020) found a trend towards greater benefit with active tDCS, and Kasashima‐Shindo et al. (2015) reported longer‐lasting (3 months) effects in the active tDCS group. Conversely, although the remaining four MI‐BCI studies also found improvements in motor performance with both active and sham tDCS, no significant differences between groups at any timepoint post intervention were observed (Ang et al. 2015; Hong et al. 2017; Hu et al. 2021; Qi et al. 2019).

## 4 Discussion

### 4.1 Synergistic Effects of Combined MIT + tDCS

This systematic review found some evidence that combined MIT and tDCS has the potential to enhance motor performance compared to MIT alone. In studies with healthy adults, improved outcomes were observed with anodal tDCS applied over the M1 simultaneously with MIT, for tasks including handwriting (Foerster et al. 2013) and finger tapping (Saimpont et al. 2016) and sequence learning (Debarnot et al. 2019). While most studies focused on upper body movements, positive effects of anodal tDCS over M1 were also observed for a postural control task (Saruco et al. 2017, 2018). In stroke patients, clinically significant improvements in upper limb motor performance were observed when anodal tDCS was applied over the affected M1 during MIT in one study (Kashoo et al. 2022). However, a positive effect of MIT alone on pain severity was reported in one study (Lagueux et al. 2018), and MIT alone was associated with improved motor performance in several studies with healthy participants (e.g., Metais et al. 2022; Muller et al. 2024; Sobierajewicz et al. 2019), with the addition of tDCS not significantly enhancing these effects.

It has been suggested that the mechanism underlying the synergistic effects of MIT with tDCS involves increased neuronal firing during MIT, induced by enhanced excitability of the primary motor cortex (M1) by tDCS (Cabral et al. 2015; Foerster et al. 2013). This enhanced excitability may also facilitate activity‐dependent neuroplastic changes, such as long‐term potentiation (LTP)‐like effects (Agboada et al. 2020; Kronberg et al. 2020). The concurrent engagement of motor networks through imagery and cortical modulation may promote Hebbian‐like plasticity, reinforcing task‐relevant neural circuits. Moreover, evidence suggests that tDCS may influence functional connectivity between motor and cognitive control regions (Grami et al. 2022). Synergistic effects appear especially relevant for task‐specific learning (Debarnot et al. 2019; Saucedo Marquez et al. 2013). In the context of BCI, the enhancement of MI signals through tDCS may increase the accuracy of feedback based on signal detection, contributing to more effective training (Chew et al. 2020). However, the present review did not find clear evidence for enhanced behavioral outcomes of combined tDCS with MIT in the context of BCI protocols for stroke patients, with only two studies indicating some enhancement of beneficial effects with active tDCS, demonstrating a trend for greater benefit compared to sham stimulation (Chew et al. 2020) and longer‐lasting benefits compared to no stimulation (Kasashima‐Shindo et al. 2015).

### 4.2 Methodological Considerations in Combined Intervention Studies

Given the variability in results of the included studies, factors that may have contributed to the different outcomes, as well as limitations of the present review, are discussed below.

In many studies, the effects of tDCS were not tested in isolation (e.g., Saruco et al. 2017, 2018), so it is unclear whether the effects of MIT combined with tDCS would be superior to tDCS alone. It can also be challenging to isolate the effects of MIT from the influence of physical practice. While one study (Debarnot et al. 2019) found greater enhancement of motor learning by tDCS for MIT than for physical practice, in other studies, tDCS was combined with MIT alongside physical training (Kashoo et al. 2022) or occupational therapy (Kasashima‐Shindo et al. 2015), which could have influenced outcomes.

Parameters of tDCS interventions, such as current intensity, electrode size, and stimulation duration may influence efficacy (Kekic et al. 2016), and heterogeneity among tDCS protocols and outcome measures presents a challenge to advancing understanding in the field (Buch et al. 2017). Most of the included studies adhered to previously established methods for administering tDCS in stroke (e.g., Kashoo et al. 2022) and pain syndrome patients (Lagueux et al. 2018). In studies with patients, intervention and session durations were longer than studies in nonpatient populations (e.g., MI‐BCI studies involved 10 sessions while non‐BCI studies typically involved 1–3 sessions); however, within the broader context of stroke rehabilitation, the training periods could be considered relatively short (Bornheim et al. 2022; Chew et al. 2020). In addition, the majority of studies with healthy individuals applied concurrent MIT and tDCS for 13–15 min for a small number of sessions, which may be insufficient to facilitate motor learning (Debarnot et al. 2019; Nikolin et al. 2018). Nonetheless, the optimal durations of combined MIT and tDCS for different populations remain undetermined and warrant further investigation.

In most studies, M1 was the primary target for stimulation, based on prior evidence linking MIT (Hardwick et al. 2018) and tDCS (Woods et al. 2016) induced motor effects to this brain area. One study also found beneficial effects from stimulation of the DLPFC (Foerster et al. 2013), which has been associated with cognitive processes in MI (e.g., Martel and Glover 2023), but the effects of tDCS on areas other than M1 have been relatively less explored (Stagg et al. 2018).

The timing of tDCS can also influence efficacy, with research suggesting that concurrent tDCS may maximize the overlap between stimulation‐induced and task‐specific neural processes, boosting performance compared to tDCS applied before training (priming), which in some cases may even diminish motor learning (Sriraman et al. 2014). One study in the present review compared timings of tDCS administration and found no significant effects of tDCS before MIT compared to tDCS during MIT (Saruco et al. 2018). Additionally, administering tDCS after motor training is suggested to be less effective, as the window of heightened neural plasticity induced, in this case by MIT, could have already passed, potentially limiting the consolidation of motor skills (Cabral et al. 2015). Therefore, concurrent tDCS with MIT may be the most beneficial combination. Nonetheless, some research suggests that priming can enhance neuroplasticity, making subsequent training more effective (Hurley and Machado 2017). Optimal timing of tDCS may also depend on the nature of the study; for example, it is challenging to administer tDCS concurrently with BCI (Hong et al. 2017). It is also important to note that BCI studies (which used tDCS prior to MIT) included in the present review were performed with stroke patients, whereas most non‐BCI studies (where tDCS was applied concurrently with MIT) were performed with healthy individuals. This difference in populations makes it difficult to directly compare motor performance outcomes between studies utilizing tDCS prior to or during MIT.

Additionally, there is some evidence that sham stimulation may induce unexpected neurobiological effects (Fonteneau et al. 2019), which could potentially account for the lack of observed differences between sham and active stimulation in some cases. Moreover, some studies did not include sham control conditions (e.g., Hu et al. 2021; Kasashima‐Shindo et al. 2015). Future research would benefit from the inclusion of conditions comparing tDCS only, MIT only, and active and sham tDCS with MIT to provide more comprehensive insights.

Some studies that yielded different results (e.g., Foerster et al. 2013; Saimpont et al. 2016; Sobierajewicz et al. 2019) used similar tDCS parameters such as electrode placement and current intensity. It is therefore possible that the combined effects of tDCS and MIT are also influenced by the nature or quality of MIT. Most of the studies in the present review found positive effects of MIT alone; however, different MIT protocols may result in different outcomes. For example, kinesthetic MI is found to activate motor areas of the brain to a greater extent than visual MI (Guillot et al. 2009). Most studies incorporated both visual and kinesthetic modalities of MI, with a smaller number utilizing visual MI only (Kashoo et al. 2022) or kinesthetic MI only (Saruco et al. 2017, 2018; Sobierajewicz et al. 2019). Other aspects of MIT interventions, such as perspectives, instructions, and engagement/adherence, can also vary between studies (see Moreno‐Verdú et al. 2024), and these factors should be considered in future research.

A further limitation of the included studies is that few achieved large samples, and the absence of significant effects in some studies could be due to the small sample size (e.g., Ang et al. 2015; Chew et al. 2020).

Individual anatomical features of the head and brain, as well as other biological and lifestyle factors, may influence variability in behavioral outcomes of tDCS (Sobierajewicz et al. 2019). Additionally, although anodal tDCS can be effective for stroke patients, the effectiveness may be greater for those with milder paresis (Hummel et al. 2005). Some participants in the included studies presented with severe levels of paresis (e.g., Kasashima‐Shindo et al. 2015), which may have limited the effects of stimulation. Nonetheless, Chew et al. (2020) reported motor improvements in patients with moderate to severe impairments, although this may be attributable to MI‐BCI training alone, since improvements were observed in both sham and active tDCS groups. There is also evidence suggesting that performance gains following tDCS may be greater in biological females (Weller et al. 2023), and that modulation of stimulation may vary according to age (Saldanha et al. 2020). Equal gender distributions were typically not achieved in the reviewed studies, and effects of age were not typically examined, although one study that included age as a covariate (Lagueux et al. 2018) did not find any significant effects of age on the results. Future work should thus include more balanced samples and examine the potential influences of age and sex on outcomes of combined MIT and tDCS. Medication is another factor that appears to interfere with responses to brain stimulation (McLaren et al. 2018). Therefore, certain drugs may have contributed to diminished effects of tDCS in the reviewed studies (Lagueux et al. 2018), but this was not consistently accounted for. In some studies with stroke patients, those who had recently used anticonvulsants were assigned to control groups without active stimulation (e.g., Kasashima‐Shindo et al. 2015), and in others, participants taking neuropsychiatric drugs were excluded (e.g., Muller et al. 2024), but medication effects were not controlled for in patients with pain syndrome (Lagueux et al. 2018).

A further consideration is that, although MIT alone was found to provide benefits in most of the included studies, the influence of MIT on motor performance could largely depend on individual differences in MI ability. The ability to effectively engage in MI may be impacted by cognitive fatigue and impairment, especially in patients and older populations (Attallah et al. 2020; Seebacher et al. 2023). MI ability was examined through self‐report measures of MI vividness in several of the included studies, usually as a screening tool to ensure that participants could engage in training (e.g., Foerster et al. 2013; Kashoo et al. 2022). One study also examined correlations between general and task‐specific MI vividness and changes in performance, but found no significant relationships (Metais et al. 2022). Nonetheless, future studies would benefit from analyzing the correspondence between performance outcomes and MI ability. Additionally, participants' previous experience with using MI (e.g., in sports or other activities) was not reported in the reviewed studies. Previous experience can influence the use of MI strategies (Bek et al. 2025) and is an important consideration in interpreting the results of MI training (Moreno‐Verdú et al. 2024).

Although the present review focused on behavioral outcomes, studies investigating the use of MI within BCI protocols have reported that neurophysiological outcomes were enhanced with the addition of tDCS, such as increased cortical excitability and modulation of neural signals, even in the absence of observable motor improvements (Chew et al. 2020; Hu et al. 2021; for review, see Mehrpour et al. 2024). In the reviewed studies, behavioral outcomes of MI‐BCI were measured using standardized clinical tests, which may not be as closely aligned with the tasks practiced during MIT as in the nonclinical studies, where greater evidence for synergistic benefits was found. Neurophysiological outcomes should thus be considered alongside behavioral measures to better understand the efficacy and mechanisms of combined MIT and tDCS.

Aside from the issues outlined above relating to the reviewed studies, it is also important to consider wider issues affecting systematic reviews and meta‐analyses. In particular, the issue of publication bias is well established (e.g., Easterbrook et al., 1991; Thornton and Lee, 2000) whereby null results are less likely to be published than significant results, which could be especially problematic when such significant results are produced by studies with small sample sizes.

### 4.3 Study Quality and Safety

The majority of included studies were RCTs, which are considered the gold standard for high‐quality clinical research (e.g., Zabor et al. 2020). However, the review also included nonrandomized and pseudo‐randomized trials, potentially compromising the overall quality of the evidence. Additionally, while many studies utilized a double‐blind design, only some tested the success of blinding and excluded participants who correctly identified active versus sham stimulation conditions (e.g., Debarnot et al. 2019). Others did not employ double blinding or verify the effectiveness of blinding (e.g., Lagueux et al. 2018), thereby increasing the risk of bias. Allocation concealment was not achieved by most studies, which is critical for reducing selection bias (Schulz and Grimes 2002). Despite these limitations, no study was rated a score below 5 on the PEDro scale, reflecting at least moderate quality across studies. Mild side effects of tDCS were reported only in two publications (Kashoo et al. 2022; Lagueux et al. 2018). Nonetheless, ethical and safety challenges persist concerning tDCS (Salehinejad et al. 2021), and many studies did not report on tDCS side effects.

### 4.4 Conclusions and Considerations for Further Research

Overall, the present review identifies some evidence of beneficial effects of combining concurrent anodal tDCS over M1 with MIT in healthy individuals. Stimulation of the affected M1 prior to MI‐BCI training in stroke patients may also enhance outcomes, but this requires further investigation. Nonetheless, several studies reported beneficial effects of MIT alone, with tDCS not significantly augmenting the effects of training. These mixed findings discourage strong conclusions about the efficacy of tDCS to augment MIT, as does a consideration of publication bias, which is relevant to any systematic review.

The review highlights the heterogeneous nature of research in this area and the considerable variability in protocols and procedures utilized, leading to the following recommendations. Further high‐quality studies, with larger sample sizes and longer intervention durations and follow‐up periods, are needed. It is also important to consider the influence of factors such as age and sex differences, and individual variation in MI ability, on treatment efficacy. Additionally, further research should investigate the optimal parameters of combined MIT and tDCS interventions, such as duration of training, stimulation targets and timing, and MI protocols (modality, instructions, and measurement). To facilitate interpretation and reproducibility, guidelines for reporting tDCS (Buch et al. 2017) and MIT (Moreno‐Verdú et al. 2024) protocols should be followed. Both behavioral and neurophysiological outcomes should be measured to increase understanding of the neural mechanisms of combined MIT and tDCS.

## Author Contributions

**Judith Bek:** conceptualization, investigation, methodology, supervision, validation, writing – original draft, writing – review and editing. **Amna Aziz:** data curation, investigation, methodology, visualization, writing – original draft, writing – review and editing. **Nuala Brady:** conceptualization, investigation, methodology, supervision, validation, writing – original draft, writing – review and editing.

## Conflicts of Interest

The authors declare no conflicts of interest.

## Peer Review

The peer review history for this article is available at https://www.webofscience.com/api/gateway/wos/peer‐review/10.1111/ejn.70280.

## Supporting information


**Data S1:** Supporting information.

## Data Availability

No primary data were generated in this study. The review was registered on PROSPERO (https://www.crd.york.ac.uk/PROSPERO/view/CRD42024533710). Search strings and data extraction templates are provided in the supplementary materials.
