# Supplementary material for: Transcranial Direct Current Stimulation to Augment Motor Imagery Training: A Systematic Review
Source: Eur J Neurosci. 2025 Oct 28;62(8):e70280. doi: 10.1111/ejn.70280 (PMC12568755; doi:10.1111/ejn.70280)
Supplement: Supplementary file 1 — Data S1: Supporting information. [file EJN-62-0-s001.pdf]

Supplementary Materials

Table A Search Strings for PUBMED

**PUBMED**
((“Transcranial Direct Current Stimulation” [Title/Abstract] OR tDCS [Title/Abstract] OR “Transcranial DC Stimulation” [Title/Abstract] OR “Direct Current Stimulation” [Title/Abstract])<br>AND<br>(“Motor Imagery” [Title/Abstract] OR “Mental Practice” [Title/Abstract] OR “Mental Rehearsal” [Title/Abstract] OR “Mental Simulation” [Title/Abstract] OR “Mental Imag*”[Title/Abstract] OR “Action Imagery” [Title/Abstract] OR “Motor Simulation” [Title/Abstract] OR “Imagery Practice” [Title/Abstract] OR “Kinaesthetic Imagery” [Title/Abstract] OR “Kinesthetic Imagery” [Title/Abstract] OR visualisation [Title/Abstract] OR visualization [Title/Abstract])<br>AND<br>(Intervention [Title/Abstract] OR Training [Title/Abstract] OR Rehabilitation [Title/Abstract] OR Recovery [Title/Abstract] OR Learning [Title/Abstract] OR Neurorehabilitation [Title/Abstract] OR Practice [Title/Abstract]))
**Web of Science**<br>(“Transcranial Direct Current Stimulation” OR tDCS OR “Transcranial DC Stimulation” OR “Direct Current Stimulation”)<br>AND<br>(“Motor Imagery” OR “Mental Practice” OR “Mental Rehearsal” OR “Mental Simulation” OR “Mental Imag*” OR “Action Imagery” OR “Motor Simulation” OR “Imagery Practice” OR “Kinaesthetic Imagery” OR “Kinesthetic Imagery” OR visualisation OR visualization)<br>AND

(Intervention OR Training OR Rehabilitation OR Recovery OR Learning OR Neurorehabilitation OR Practice)

Embase

('Transcranial Direct Current Stimulation':ti,ab OR tDCS:ti,ab OR 'Transcranial DC Stimulation':ti,ab OR 'Direct Current Stimulation':ti,ab)

AND

('Motor Imagery':ti,ab OR 'Mental Practice':ti,ab OR 'Mental Rehearsal':ti,ab OR 'Mental Simulation':ti,ab OR 'Mental Imag*':ti,ab OR 'Action Imagery':ti,ab OR 'Motor Simulation':ti,ab OR 'Imagery Practice':ti,ab OR 'Kinaesthetic Imagery':ti,ab OR 'Kinesthetic Imagery':ti,ab OR visualisation:ti,ab OR visualization:ti,ab)

AND

(Intervention:ti,ab OR Training:ti,ab OR Rehabilitation:ti,ab OR Recovery:ti,ab OR Learning:ti,ab OR Neurorehabilitation:ti,ab OR Practice:ti,ab)

ProQuest

(“Transcranial Direct Current Stimulation” OR tDCS OR “Transcranial DC Stimulation” OR “Direct Current Stimulation”)

AND

(“Motor Imagery” OR “Mental Practice” OR “Mental Rehearsal” OR “Mental Simulation” OR “Mental Imag*” OR “Action Imagery” OR “Motor Simulation” OR “Imagery Practice” OR “Kinaesthetic Imagery” OR “Kinesthetic Imagery” OR visualisation OR visualization)

AND

(Intervention OR Training OR Rehabilitation OR Recovery OR Learning OR Neurorehabilitation OR Practice)

Ab (“Transcranial Direct Current Stimulation” OR tDCS OR “Transcranial DC Stimulation” OR “Direct Current Stimulation”) AND Ab (“Motor Imagery” OR “Mental Practice” OR “Mental Rehearsal” OR “Mental Simulation” OR “Mental Imag*” OR “Action Imagery” OR “Motor Simulation” OR “Imagery Practice” OR “Kinaesthetic Imagery” OR “Kinesthetic Imagery” OR visualisation OR visualization) AND Ab (Intervention OR Training OR Rehabilitation OR Recovery OR Learning OR Neurorehabilitation OR Practice)

Table B PEDro scores for the included studies

	Q1	Q2	Q3	Q4	Q5	Q6	Q7	Q8	Q9	Q 10	Q 11	Total
Ang et al. (2015)	1	1	0	1	0	0	0	1	1	1	1	6
Chew et al. (2020)	1	1	1	1	1	0	1	1	1	1	1	9
Debarnot et al., (2019)	1	1	0	1	1	1	1	1	1	1	1	9
Foerster et al. (2013)	1	1	0	0	1	0	1	1	1	1	0	6
Hong et al. (2017)	1	1	0	1	1	0	1	1	1	1	0	7
Hu et al. (2021)	1	1	0	1	1	0	0	1	1	1	1	7
Kasashima-Shindo et al. (2015)	1	0	0	1	1	0	0	1	1	1	1	6
Kashoo et al. (2022)	1	1	1	1	1	0	1	1	1	1	1	9
Lagueux et al. (2018)	1	1	1	1	1	0	0	1	1	1	1	8
Metais et al. (2022)	1	1	0	1	1	1	1	1	1	1	1	9
Muller et al. (2024)	1	1	0	1	1	1	1	1	1	1	1	9
Qi et al. (2019)	0	1	0	1	1	1	1	1	1	1	1	8
Saimpont et al. (2016)	1	1	0	1	1	1	1	1	1	1	1	9
Saruco et al. (2017)	1	1	0	0	1	1	1	1	1	1	1	8
Saruco et al. (2018)	1	1	0	0	1	1	1	1	1	1	1	8
Sobierajewicz et al., (2019)	1	1	0	0	1	0	0	1	1	1	0	5

*Q1: This criterion influences external validity, but not the internal or statistical validity of the trial.

Therefore, this item is not used to calculate the final PEDro score.

Questions included in the PEDro scale

Q1- Eligibility criteria were specified

Q2- Subjects were randomly allocated to groups (in a crossover study, subjects were randomly allocated an order in which treatments were received)

Q3- Allocation was concealed

Q4- The groups were similar at baseline regarding the most important prognostic indicators

Q5- There was blinding of all subjects

Q6- There was blinding of all therapists who administered the therapy

Q7- There was blinding of all assessors who measured at least one key outcome

Q8- Measures of at least one key outcome were obtained from more than 85% of the subjects initially allocated to groups

Q9- All subjects for whom outcome measures were available received the treatment or control condition as allocated or, where this was not the case, data for at least one key outcome was analysed by "intention to treat"

Q10- The results of between-group statistical comparisons are reported for at least one key outcome

Q11- The study provides both point measures and measures of variability for at least one key outcome

Data extraction template

STUDY TITLE/ AUTHOR/YEAR	STUDY/EXPERIMENTAL DESIGN	AIMS/OBJECTIVES	SAMPLE SIZE	INCLUSION/EXCLUSION CRITERIA	AGE RANGE	GENDER	HEALTH STATUS	INTERVENTION TYPE	PRE/POST INTERVENTION	tDCS DETAILS	MI DETAILS / INSTRUCTIONS	COMBINED INTERVENTIONAL PROTOCOL

Data extraction template (continued)

OTHER INTERVENTION DETAILS	COMPARATOR/ CONTROL DETAILS	OUTCOMES MEASURES	MEASUREMENT TOOLS	STATISTICAL SIGNIFICANCE	OUTCOMES RESULTS/CONCLUSIONS	RISK OF BIAS (BLINDING/ RANDOMISATION	PEDro/Cochrane RoB Scale Total Score/STUDY QUALITY	STRENGHTS/LIMITATIONS/FUTURE DIRECTIONS	ADVERSE EFFECTS	IMPLICATIONS	ADDITIONAL COMMENTS
